# Supplementary material for: Open access-enabled evaluation of epigenetic age acceleration in colorectal cancer and development of a classifier with diagnostic potential
Source: Front Genet. 2023 Oct 24;14:1258648. doi: 10.3389/fgene.2023.1258648 (PMC10634722; doi:10.3389/fgene.2023.1258648)
Supplement: Supplementary file 1 [file Image1.pdf]

Supplementary Material

1 SUPPLEMENTARY TABLES AND FIGURES

1.1 Supplementary Tables

Table S1. List of datasets used in this study and sample distribution in each dataset.

Dataset ID	Platform	Dataset 1				Dataset 2	
		Healthy	Normal	Tumour	Adenoma	Healthy	Normal
E-MTAB-7036	450K	0	27	189	0	0	27
E-MTAB-7854	EPIC	0	0	0	64	0	0
E-MTAB-3027	450K	0	23	23	0	0	23
GSE101764	450K	0	144	103	0	0	144
GSE131013	450K	48	96	93	0	48	94
GSE132804	450K	73	20	0	0	73	20
GSE132804	EPIC	151	49	0	0	151	46
GSE142257	EPIC	118	0	0	0	118	0
GSE149282	EPIC	0	10	10	0	0	9
GSE151732	EPIC	254	0	0	0	253	0
GSE159898	EPIC	0	16	22	0	0	14
GSE166212	EPIC	0	6	24	8	0	5
GSE171550	EPIC	0	52	0	0	0	47
GSE199057	EPIC	72	79	71	0	72	76

Table S2. Sample distribution in datasets used for classifier.

Dataset	Healthy	Normal	Platform	Test/Train
E-MTAB-7036	0	24	450k	Test
E-MTAB-3027	0	22	450k	Test
GSE101764	0	140	450k	Train
GSE132804	73	17	450k	Train
GSE132804	150	44	EPIC	Train
GSE142257	118	0	EPIC	Train
GSE149282	0	9	EPIC	Train
GSE151732	250	0	EPIC	Test
GSE166212	0	5	EPIC	Train
GSE199057	71	67	EPIC	Test

Table S3. Summary of epigenetic age (EA) data. p-values obtained from Welch's t-test, testing the difference between male and female groups for each EA ($H0$: mean value of the EA is the same for male and female groups). Significant differences ($p < 0.05$) are highlighted with bold text.

Clock	Tissue	Mean (SD)			p-value	95% CI
		All	Female	Male		
**Horvath**	Healthy	62.12 (7.74)	61.55 (7.56)	63.1 (7.97)	**0.011**	(-2.75, -0.36)
	Normal	63.61 (9.58)	62.86 (9.57)	64.1 (9.57)	0.148	(-2.92, 0.44)
	Tumour	55.48 (14.14)	54.91 (14.84)	55.91 (13.6)	0.427	(-3.45, 1.46)
	Adenoma	67.6 (9.91)	68.71 (9.35)	65.36 (10.81)	0.202	(-1.87, 8.58)
**Hannum**	Healthy	66.63 (9.19)	65.92 (8.95)	67.86 (9.49)	**0.007**	(-3.35, -0.52)
	Normal	67.28 (18.35)	65.94 (18.24)	68.15 (18.4)	0.178	(-5.44, 1.01)
	Tumour	72.45 (17.77)	73.73 (18.7)	71.49 (17.02)	0.155	(-0.85, 5.33)
	Adenoma	83.04 (11.45)	84.43 (9.41)	80.24 (14.55)	0.208	(-2.45, 10.84)
**PhenoAge**	Healthy	62.84 (10.41)	61.84 (10.48)	64.57 (10.06)	**0.001**	(-4.28, -1.17)
	Normal	66.34 (20.3)	65.57 (21.06)	66.84 (19.81)	0.491	(-4.89, 2.35)
	Tumour	92.93 (26.37)	95.05 (29.19)	91.35 (23.97)	0.119	(-0.96, 8.34)
	Adenoma	136.16 (21.11)	137.17 (18.93)	134.13 (25.24)	0.606	(-8.79, 14.86)
**SkinBlood**	Healthy	70.82 (8.63)	70.73 (8.7)	70.98 (8.51)	0.715	(-1.55, 1.06)
	Normal	69.61 (12.29)	69.14 (13.06)	69.92 (11.77)	0.486	(-3, 1.43)
	Tumour	81.26 (18.32)	82.6 (19.32)	80.26 (17.5)	0.15	(-0.85, 5.52)
	Adenoma	102.44 (11.49)	104.74 (10.79)	97.82 (11.66)	**0.019**	(1.18, 12.66)
**PedBE**	Healthy	13.72 (1.71)	13.68 (1.69)	13.79 (1.76)	0.422	(-0.37, 0.16)
	Normal	12.71 (2.1)	12.69 (2.1)	12.73 (2.11)	0.846	(-0.41, 0.33)
	Tumour	12.47 (2.8)	12.35 (2.92)	12.55 (2.71)	0.424	(-0.68, 0.29)
	Adenoma	15.21 (2.48)	15.28 (2.22)	15.07 (2.97)	0.767	(-1.19, 1.6)
**Wu**	Healthy	11.53 (1)	11.47 (0.97)	11.65 (1.03)	**0.021**	(-0.33, -0.03)
	Normal	9.73 (2.53)	9.65 (2.43)	9.78 (2.59)	0.571	(-0.57, 0.31)
	Tumour	11.82 (2.02)	11.71 (1.87)	11.91 (2.12)	0.23	(-0.55, 0.13)
	Adenoma	12.11 (1.63)	11.86 (1.25)	12.6 (2.14)	0.124	(-1.71, 0.22)
**Zhang BLUP**	Healthy	73.81 (7.68)	73.47 (7.76)	74.4 (7.53)	0.115	(-2.09, 0.23)
	Normal	71.32 (12.38)	71.17 (13.07)	71.41 (11.93)	0.829	(-2.47, 1.98)
	Tumour	82.54 (15.55)	84.14 (16.76)	81.34 (14.5)	**0.044**	(0.08, 5.52)
	Adenoma	109.19 (9.71)	108.48 (8.72)	110.6 (11.52)	0.432	(-7.53, 3.29)
**Zhang EN**	Healthy	75.82 (7.45)	75.46 (7.52)	76.44 (7.31)	0.085	(-2.11, 0.14)
	Normal	74.37 (10.83)	73.84 (11.12)	74.72 (10.64)	0.368	(-2.81, 1.04)
	Tumour	76.67 (11.69)	77.54 (12.39)	76.01 (11.11)	0.141	(-0.51, 3.56)
	Adenoma	98.61 (7.33)	99.24 (6.53)	97.37 (8.73)	0.36	(-2.22, 5.96)
**EpiTOC**	Healthy	0.17 (0.04)	0.17 (0.04)	0.17 (0.04)	0.954	(-0.01, 0.01)
	Normal	0.17 (0.07)	0.16 (0.07)	0.17 (0.07)	0.534	(-0.02, 0.01)
	Tumour	0.3 (0.09)	0.31 (0.1)	0.29 (0.08)	**0.003**	(0.01, 0.04)
	Adenoma	0.4 (0.07)	0.41 (0.06)	0.39 (0.08)	0.376	(-0.02, 0.05)
**HypoClock**	Healthy	0.86 (0.02)	0.86 (0.02)	0.86 (0.02)	**0.035**	(0, 0.01)
	Normal	0.84 (0.06)	0.84 (0.06)	0.84 (0.07)	0.33	(-0.01, 0.02)
	Tumour	0.72 (0.09)	0.72 (0.09)	0.72 (0.09)	0.568	(-0.02, 0.01)
	Adenoma	0.72 (0.08)	0.74 (0.07)	0.69 (0.08)	**0.01**	(0.01, 0.09)
**MiAge**	Healthy	1990.07 (512.67)	1998.84 (492.43)	1974.98 (546.42)	0.56	(-56.45, 104.16)
	Normal	1708.7 (1032.78)	1691.57 (1129.02)	1719.87 (966.6)	0.767	(-216.3, 159.72)
	Tumour	3942.91 (1747.98)	4232.13 (1832.32)	3726.46 (1652.25)	**0.001**	(203.9, 807.45)
	Adenoma	5484.91 (1522.72)	5339.43 (1437.43)	5775.87 (1674.18)	0.281	(-1244.2, 371.32)

Table S4. Summary of epigenetic age acceleration (EAA) scores (sex-adjusted) from Dataset 1. p-values obtained from Welch's t-test, testing the difference between male and female groups for each EAA ($H0$: mean value of the EAA is the same for male and female groups). Significant differences ($p < 0.05$) are highlighted with bold text.

EAA	Tissue	Mean (SD)			p-value	95% CI
		All	Female	Male		
**Horvath AA**	Healthy	0.593 (4.351)	0.691 (4.186)	0.423 (4.625)	0.439	(-0.412, 0.949)
	Normal	3.481 (5.84)	3.91 (5.844)	3.201 (5.829)	0.176	(-0.319, 1.736)
	Tumour	-4.19 (13.488)	-4.17 (14.321)	-4.206 (12.853)	0.976	(-2.318, 2.391)
	Adenoma	0.006 (10.224)	-0.654 (9.966)	1.327 (10.816)	0.456	(-7.296, 3.333)
**Hannum AA**	Healthy	1.077 (4.865)	1.134 (4.962)	0.978 (4.7)	0.674	(-0.574, 0.887)
	Normal	-1.39 (10.057)	-0.771 (9.96)	-1.793 (10.115)	0.256	(-0.742, 2.785)
	Tumour	-0.014 (11.892)	2.278 (11.928)	-1.729 (11.59)	**<0.001**	(1.985, 6.031)
	Adenoma	-0.53 (8.013)	-0.295 (7.376)	-1.001 (9.311)	0.747	(-3.704, 5.117)
**Pheno AA**	Healthy	-0.169 (7.902)	0.56 (7.795)	-1.424 (7.943)	**0.001**	(0.782, 3.185)
	Normal	-10.45 (15.836)	-9.46 (15.751)	-11.095 (15.882)	0.248	(-1.145, 4.417)
	Tumour	10.327 (24.186)	12.405 (26.268)	8.771 (22.421)	0.093	(-0.607, 7.874)
	Adenoma	0.71 (18.79)	-0.518 (19.028)	3.164 (18.455)	0.434	(-13.059, 5.694)
**SkinBlood AA**	Healthy	-0.178 (5.188)	-0.215 (5.297)	-0.114 (5.002)	0.798	(-0.88, 0.677)
	Normal	-3.661 (7.933)	-3.928 (8.158)	-3.486 (7.791)	0.538	(-1.853, 0.968)
	Tumour	3.649 (15.474)	3.892 (16.3)	3.467 (14.849)	0.757	(-2.27, 3.12)
	Adenoma	1.194 (10.834)	1.437 (11.262)	0.708 (10.139)	0.783	(-4.555, 6.013)
**PedBE AA**	Healthy	0.126 (0.85)	0.112 (0.837)	0.149 (0.873)	0.572	(-0.169, 0.093)
	Normal	0.022 (1.209)	0.07 (1.217)	-0.01 (1.205)	0.465	(-0.134, 0.293)
	Tumour	-0.213 (1.948)	-0.325 (2.034)	-0.128 (1.881)	0.253	(-0.535, 0.141)
	Adenoma	0.174 (1.848)	0.008 (1.751)	0.506 (2.024)	0.31	(-1.476, 0.481)
**Wu AA**	Healthy	0.172 (0.779)	0.118 (0.749)	0.264 (0.822)	**0.018**	(-0.267, -0.025)
	Normal	-1.02 (2.231)	-1.012 (2.188)	-1.025 (2.262)	0.944	(-0.376, 0.404)
	Tumour	0.769 (1.934)	0.633 (1.88)	0.871 (1.971)	0.157	(-0.567, 0.092)
	Adenoma	-0.028 (1.588)	-0.214 (1.259)	0.344 (2.08)	0.236	(-1.499, 0.384)
**Zhang BLUP AA**	Healthy	0.218 (4.833)	0.237 (4.793)	0.185 (4.91)	0.889	(-0.689, 0.794)
	Normal	-4.053 (9.205)	-3.712 (9.27)	-4.275 (9.169)	0.497	(-1.062, 2.187)
	Tumour	3.495 (12.305)	4.194 (13.087)	2.971 (11.68)	0.263	(-0.923, 3.371)
	Adenoma	1.248 (9.155)	-0.766 (8.104)	5.278 (9.955)	**0.014**	(-10.788, -1.3)
**Zhang EN AA**	Healthy	0.624 (4.462)	0.644 (4.501)	0.591 (4.404)	0.878	(-0.623, 0.729)
	Normal	-0.784 (6.521)	-0.564 (6.577)	-0.927 (6.491)	0.537	(-0.789, 1.514)
	Tumour	-0.208 (8.422)	0.444 (8.698)	-0.696 (8.19)	0.125	(-0.317, 2.597)
	Adenoma	1.019 (6.152)	0.201 (5.713)	2.655 (6.777)	0.135	(-5.709, 0.801)
**EpiTOC AA**	Healthy	-0.005 (0.036)	-0.005 (0.034)	-0.005 (0.039)	0.96	(-0.006, 0.006)
	Normal	-0.051 (0.054)	-0.054 (0.055)	-0.049 (0.053)	0.321	(-0.014, 0.005)
	Tumour	0.056 (0.088)	0.062 (0.092)	0.052 (0.085)	0.179	(-0.005, 0.026)
	Adenoma	0.004 (0.064)	0.002 (0.059)	0.007 (0.075)	0.814	(-0.039, 0.031)
**HypoClock AA**	Healthy	0.006 (0.021)	0.003 (0.019)	0.011 (0.024)	**<0.001**	(-0.011, -0.004)
	Normal	0.049 (0.045)	0.051 (0.046)	0.047 (0.045)	0.329	(-0.004, 0.012)
	Tumour	-0.054 (0.092)	-0.052 (0.094)	-0.056 (0.09)	0.694	(-0.013, 0.019)
	Adenoma	-0.009 (0.065)	0.004 (0.059)	-0.033 (0.071)	**0.036**	(0.003, 0.071)
**MiAge AA**	Healthy	-72.183 (399.83)	-87.704 (385.32)	-45.448 (423.077)	0.184	(-104.643, 20.13)
	Normal	-826.846 (929.711)	-878.407 (1005.168)	-793.233 (877.051)	0.321	(-253.595, 83.246)
	Tumour	891.199 (1608.919)	1013.286 (1627.703)	799.833 (1591.255)	0.13	(-63.337, 490.243)
	Adenoma	90.346 (1437.475)	-19.894 (1404.599)	310.826 (1506.991)	0.374	(-1073.536, 412.097)

Table S5. Summary of epigenetic age acceleration (EAA) scores (unadjusted) from Dataset 1. p-values obtained from Welch's t-test, testing the difference between male and female groups for each EAA ($H0$: mean value of the EAA is the same for male and female groups). Significant differences ($p < 0.05$) are highlighted with bold text.

EAA	Tissue	Mean (SD)			p-value	95% CI
		All	Female	Male		
**Horvath AA**	Healthy	0.565 (4.336)	0.303 (4.153)	1.016 (4.607)	**0.039**	(-1.39, -0.036)
	Normal	3.489 (5.83)	3.251 (5.848)	3.645 (5.822)	0.451	(-1.422, 0.633)
	Tumour	-4.163 (13.523)	-4.765 (14.351)	-3.712 (12.873)	0.381	(-3.412, 1.306)
	Adenoma	0.018 (10.296)	-1.005 (9.986)	2.063 (10.813)	0.251	(-8.385, 2.248)
**Hannum AA**	Healthy	1.054 (4.869)	0.709 (4.927)	1.648 (4.717)	**0.012**	(-1.669, -0.209)
	Normal	-1.389 (10.082)	-1.48 (9.988)	-1.33 (10.158)	0.868	(-1.919, 1.62)
	Tumour	0.015 (11.881)	1.652 (12.001)	-1.21 (11.661)	**0.006**	(0.827, 4.898)
	Adenoma	-0.521 (8.115)	-0.707 (7.443)	-0.149 (9.481)	0.802	(-5.04, 3.925)
**Pheno AA**	Healthy	-0.224 (7.85)	-0.222 (7.791)	-0.227 (7.964)	0.993	(-1.198, 1.208)
	Normal	-10.438 (15.773)	-10.799 (15.688)	-10.202 (15.849)	0.672	(-3.37, 2.175)
	Tumour	10.383 (24.092)	11.2 (26.229)	9.771 (22.385)	0.508	(-2.805, 5.663)
	Adenoma	0.752 (18.921)	-1.21 (19.008)	4.675 (18.513)	0.214	(-15.278, 3.509)
**SkinBlood AA**	Healthy	-0.173 (5.188)	-0.153 (5.298)	-0.208 (5.001)	0.889	(-0.723, 0.834)
	Normal	-3.662 (7.936)	-3.823 (8.164)	-3.557 (7.795)	0.711	(-1.678, 1.146)
	Tumour	3.644 (15.479)	3.987 (16.304)	3.388 (14.853)	0.662	(-2.096, 3.295)
	Adenoma	1.19 (10.841)	1.492 (11.265)	0.587 (10.146)	0.733	(-4.382, 6.192)
**PedBE AA**	Healthy	0.125 (0.849)	0.104 (0.836)	0.16 (0.872)	0.4	(-0.187, 0.075)
	Normal	0.022 (1.208)	0.058 (1.216)	-0.002 (1.204)	0.583	(-0.154, 0.273)
	Tumour	-0.212 (1.948)	-0.336 (2.033)	-0.119 (1.88)	0.209	(-0.555, 0.122)
	Adenoma	0.174 (1.848)	0.001 (1.751)	0.52 (2.022)	0.29	(-1.497, 0.458)
**Wu AA**	Healthy	0.168 (0.785)	0.068 (0.746)	0.34 (0.821)	**<0.001**	(-0.393, -0.15)
	Normal	-1.019 (2.23)	-1.096 (2.185)	-0.969 (2.261)	0.524	(-0.516, 0.263)
	Tumour	0.773 (1.937)	0.558 (1.877)	0.934 (1.969)	**0.025**	(-0.705, -0.047)
	Adenoma	-0.025 (1.602)	-0.259 (1.261)	0.442 (2.082)	0.14	(-1.642, 0.242)
**Zhang BLUP AA**	Healthy	0.205 (4.839)	0.055 (4.794)	0.463 (4.915)	0.281	(-1.149, 0.334)
	Normal	-4.051 (9.199)	-4.022 (9.262)	-4.07 (9.171)	0.953	(-1.576, 1.672)
	Tumour	3.508 (12.302)	3.918 (13.091)	3.202 (11.688)	0.513	(-1.432, 2.864)
	Adenoma	1.262 (9.236)	-0.928 (8.113)	5.641 (9.939)	**0.008**	(-11.308, -1.829)
**Zhang EN AA**	Healthy	0.612 (4.461)	0.467 (4.491)	0.862 (4.408)	0.251	(-1.071, 0.281)
	Normal	-0.782 (6.519)	-0.86 (6.575)	-0.731 (6.493)	0.826	(-1.28, 1.023)
	Tumour	-0.196 (8.429)	0.179 (8.714)	-0.476 (8.212)	0.378	(-0.804, 2.116)
	Adenoma	1.035 (6.205)	0.043 (5.721)	3.019 (6.768)	0.072	(-6.229, 0.277)
**EpiTOC AA**	Healthy	-0.005 (0.036)	-0.005 (0.034)	-0.006 (0.039)	0.752	(-0.005, 0.007)
	Normal	-0.051 (0.054)	-0.053 (0.055)	-0.049 (0.053)	0.415	(-0.014, 0.006)
	Tumour	0.056 (0.088)	0.063 (0.092)	0.051 (0.085)	0.146	(-0.004, 0.027)
	Adenoma	0.004 (0.064)	0.003 (0.059)	0.006 (0.075)	0.852	(-0.039, 0.032)
**HypoClock AA**	Healthy	0.006 (0.021)	0.004 (0.019)	0.009 (0.024)	**0.003**	(-0.009, -0.002)
	Normal	0.049 (0.045)	0.053 (0.046)	0.046 (0.045)	0.098	(-0.001, 0.015)
	Tumour	-0.054 (0.092)	-0.051 (0.094)	-0.057 (0.09)	0.462	(-0.01, 0.022)
	Adenoma	-0.009 (0.065)	0.004 (0.059)	-0.035 (0.071)	**0.024**	(0.005, 0.073)
**MiAge AA**	Healthy	-71.386 (398.219)	-76.445 (384.038)	-62.672 (422.14)	0.664	(-75.999, 48.452)
	Normal	-827.019 (929.725)	-859.119 (1006.151)	-806.093 (877.344)	0.537	(-221.575, 115.524)
	Tumour	890.387 (1610.832)	1030.627 (1628.608)	785.436 (1591.987)	0.083	(-31.742, 522.124)
	Adenoma	89.706 (1436.211)	-9.966 (1404.747)	289.051 (1507.596)	0.422	(-1042.07, 444.037)

Table S6. Summary of epigenetic age acceleration (EAA) scores from Dataset 2. p-values obtained from Welch's t-test, testing the difference between male and female groups for each EAA ($H0$: mean value of the EAA is the same for male and female groups). Significant differences ($p < 0.05$) are highlighted with bold text.

EAA	Tissue	Mean (SD)			p-value	95% CI
		All	Female	Male		
**Sex-adjusted**						
**Horvath AA**	Healthy	-0.041 (2.692)	0.073 (2.729)	-0.237 (2.621)	0.135	(-0.096, 0.715)
	Normal	0.058 (3.106)	0.208 (3.014)	-0.039 (3.165)	0.379	(-0.304, 0.798)
**Hannum AA**	Healthy	0.496 (3.806)	0.575 (3.946)	0.359 (3.554)	0.453	(-0.348, 0.78)
	Normal	-0.702 (5.458)	-0.011 (5.238)	-1.143 (5.558)	**0.021**	(0.17, 2.094)
**Pheno AA**	Healthy	0.535 (5.048)	0.772 (5.24)	0.126 (4.68)	0.089	(-0.099, 1.393)
	Normal	-0.758 (6.878)	0.144 (6.296)	-1.335 (7.177)	**0.015**	(0.285, 2.671)
**SkinBlood AA**	Healthy	0.241 (4.461)	0.17 (4.586)	0.365 (4.242)	0.564	(-0.862, 0.471)
	Normal	-0.342 (5.712)	-0.192 (5.716)	-0.437 (5.716)	0.638	(-0.78, 1.27)
**PedBE AA**	Healthy	0.069 (0.791)	0.063 (0.787)	0.079 (0.8)	0.8	(-0.137, 0.106)
	Normal	-0.098 (0.946)	-0.049 (0.889)	-0.129 (0.98)	0.344	(-0.086, 0.246)
**Wu AA**	Healthy	0.046 (0.415)	0.035 (0.435)	0.065 (0.379)	0.334	(-0.091, 0.031)
	Normal	-0.066 (0.446)	-0.054 (0.439)	-0.073 (0.45)	0.642	(-0.061, 0.098)
**Zhang BLUP AA**	Healthy	0.323 (3.554)	0.318 (3.556)	0.332 (3.557)	0.96	(-0.556, 0.528)
	Normal	-0.457 (4.874)	0.089 (4.687)	-0.806 (4.966)	**0.041**	(0.035, 1.756)
**Zhang EN AA**	Healthy	0.508 (4.619)	0.557 (4.644)	0.423 (4.584)	0.708	(-0.568, 0.836)
	Normal	-0.72 (6.011)	-0.109 (5.784)	-1.11 (6.129)	0.065	(-0.061, 2.063)
**EpiTOC AA**	Healthy	0.001 (0.017)	0 (0.017)	0.002 (0.016)	0.36	(-0.004, 0.001)
	Normal	-0.001 (0.016)	-0.001 (0.015)	-0.002 (0.017)	0.548	(-0.002, 0.004)
**HypoClock AA**	Healthy	-0.001 (0.009)	-0.001 (0.009)	0 (0.009)	0.099	(-0.003, 0)
	Normal	0.001 (0.013)	0 (0.012)	0.002 (0.013)	0.137	(-0.004, 0.001)
**MiAge AA**	Healthy	11.357 (157.03)	3.819 (166.835)	24.392 (137.784)	0.076	(-43.297, 2.15)
	Normal	-16.08 (166.637)	-14.158 (153.926)	-17.31 (174.516)	0.831	(-25.94, 32.245)
**Unadjusted**						
**Horvath AA**	Healthy	-0.055 (2.688)	-0.116 (2.728)	0.052 (2.618)	0.415	(-0.574, 0.237)
	Normal	0.077 (3.108)	-0.139 (3.011)	0.216 (3.165)	0.206	(-0.906, 0.196)
**Hannum AA**	Healthy	0.485 (3.816)	0.327 (3.941)	0.757 (3.581)	0.137	(-0.996, 0.137)
	Normal	-0.686 (5.502)	-0.489 (5.303)	-0.812 (5.63)	0.515	(-0.652, 1.297)
**Pheno AA**	Healthy	0.524 (5.074)	0.437 (5.254)	0.676 (4.754)	0.534	(-0.993, 0.515)
	Normal	-0.742 (7.005)	-0.517 (6.444)	-0.887 (7.348)	0.553	(-0.852, 1.59)
**SkinBlood AA**	Healthy	0.247 (4.466)	0.243 (4.592)	0.254 (4.246)	0.974	(-0.678, 0.656)
	Normal	-0.35 (5.723)	-0.067 (5.725)	-0.531 (5.724)	0.375	(-0.563, 1.491)
**PedBE AA**	Healthy	0.069 (0.791)	0.06 (0.787)	0.084 (0.799)	0.697	(-0.145, 0.097)
	Normal	-0.098 (0.945)	-0.055 (0.888)	-0.125 (0.98)	0.411	(-0.096, 0.235)
**Wu AA**	Healthy	0.046 (0.416)	0.026 (0.435)	0.08 (0.379)	0.079	(-0.116, 0.006)
	Normal	-0.065 (0.446)	-0.073 (0.439)	-0.059 (0.451)	0.723	(-0.094, 0.065)
**Zhang BLUP AA**	Healthy	0.317 (3.56)	0.227 (3.558)	0.474 (3.566)	0.371	(-0.791, 0.296)
	Normal	-0.449 (4.881)	-0.078 (4.702)	-0.686 (4.985)	0.167	(-0.255, 1.471)
**Zhang EN AA**	Healthy	0.497 (4.623)	0.384 (4.642)	0.694 (4.593)	0.387	(-1.013, 0.393)
	Normal	-0.704 (6.005)	-0.4 (5.793)	-0.898 (6.138)	0.358	(-0.565, 1.561)
**EpiTOC AA**	Healthy	0.001 (0.017)	0.001 (0.017)	0.001 (0.016)	0.608	(-0.003, 0.002)
	Normal	-0.001 (0.016)	0 (0.015)	-0.002 (0.017)	0.287	(-0.001, 0.004)
**HypoClock AA**	Healthy	-0.001 (0.009)	-0.001 (0.009)	-0.001 (0.009)	0.607	(-0.002, 0.001)
	Normal	0.001 (0.013)	0.001 (0.013)	0.002 (0.013)	0.564	(-0.003, 0.002)
**MiAge AA**	Healthy	11.592 (156.914)	6.911 (166.966)	19.686 (137.746)	0.27	(-35.503, 9.954)
	Normal	-16.413 (166.859)	-8.101 (154.073)	-21.729 (174.585)	0.358	(-15.485, 42.741)

Table S7. EAA differences between four different tissue (healthy ($n = 716$), normal ($n = 522$), tumour ($n = 535$), adenoma ($n = 72$)) from Dataset 1. p-values and 95% confidence interval were obtained from two-sample t-test. Significant differences ($p < 0.05$) are highlighted with bold text.

EAA	Reference	Tissue	Sex-adjusted		Unadjusted	
			p	95% CI	p	95% CI
Horvath AA	normal	healthy	**<0.001**	(2.293, 3.482)	**<0.001**	(2.331, 3.518)
	healthy	tumour	**<0.001**	(3.594, 5.972)	**<0.001**	(3.536, 5.919)
	healthy	adenoma	0.631	(-1.836, 3.009)	0.656	(-1.892, 2.987)
	normal	tumour	**<0.001**	(6.421, 8.921)	**<0.001**	(6.400, 8.905)
	normal	adenoma	**0.006**	(1.022, 5.927)	**0.006**	(1.003, 5.941)
	tumour	adenoma	**0.002**	(-6.850, -1.543)	**0.002**	(-6.851, -1.511)
Hannum AA	normal	healthy	**<0.001**	(-3.402, -1.532)	**<0.001**	(-3.380, -1.506)
	healthy	tumour	**0.046**	(0.020, 2.162)	0.057	(-0.031, 2.109)
	healthy	adenoma	0.099	(-0.308, 3.523)	0.110	(-0.364, 3.514)
	normal	tumour	**0.042**	(-2.704, -0.048)	**0.038**	(-2.733, -0.075)
	normal	adenoma	0.411	(-2.925, 1.207)	0.412	(-2.957, 1.221)
	tumour	adenoma	0.632	(-1.613, 2.646)	0.623	(-1.614, 2.686)
Pheno AA	normal	healthy	**<0.001**	(-11.760, -8.802)	**<0.001**	(-11.686, -8.741)
	healthy	tumour	**<0.001**	(-12.629, -8.362)	**<0.001**	(-12.732, -8.482)
	healthy	adenoma	0.695	(-5.331, 3.573)	0.666	(-5.458, 3.506)
	normal	tumour	**<0.001**	(-23.238, -18.314)	**<0.001**	(-23.273, -18.368)
	normal	adenoma	**<0.001**	(-15.773, -6.546)	**<0.001**	(-15.831, -6.549)
	tumour	adenoma	**<0.001**	(4.761, 14.472)	**<0.001**	(4.751, 14.511)
SkinBlood AA	normal	healthy	**<0.001**	(-4.263, -2.702)	**<0.001**	(-4.269, -2.708)
	healthy	tumour	**<0.001**	(-5.195, -2.459)	**<0.001**	(-5.186, -2.449)
	healthy	adenoma	0.291	(-3.945, 1.201)	0.295	(-3.938, 1.211)
	normal	tumour	**<0.001**	(-8.789, -5.830)	**<0.001**	(-8.786, -5.826)
	normal	adenoma	**<0.001**	(-7.487, -2.222)	**<0.001**	(-7.486, -2.218)
	tumour	adenoma	0.091	(-0.401, 5.310)	0.092	(-0.403, 5.311)
PedBE AA	normal	healthy	0.093	(-0.225, 0.017)	0.095	(-0.224, 0.018)
	healthy	tumour	**<0.001**	(0.161, 0.515)	**<0.001**	(0.160, 0.514)
	healthy	adenoma	0.827	(-0.487, 0.390)	0.824	(-0.487, 0.389)
	normal	tumour	**0.019**	(0.039, 0.429)	**0.019**	(0.039, 0.429)
	normal	adenoma	0.499	(-0.598, 0.294)	0.499	(-0.598, 0.294)
	tumour	adenoma	0.101	(-0.850, 0.077)	0.102	(-0.850, 0.078)
Wu AA	normal	healthy	**<0.001**	(-1.392, -0.991)	**<0.001**	(-1.387, -0.987)
	healthy	tumour	**<0.001**	(-0.771, -0.424)	**<0.001**	(-0.779, -0.431)
	healthy	adenoma	0.296	(-0.178, 0.577)	0.316	(-0.188, 0.574)
	normal	tumour	**<0.001**	(-2.041, -1.537)	**<0.001**	(-2.044, -1.540)
	normal	adenoma	**<0.001**	(-1.410, -0.574)	**<0.001**	(-1.415, -0.573)
	tumour	adenoma	**<0.001**	(0.390, 1.203)	**<0.001**	(0.388, 1.208)
Zhang BLUP AA	normal	healthy	**<0.001**	(-5.138, -3.404)	**<0.001**	(-5.123, -3.390)
	healthy	tumour	**<0.001**	(-4.380, -2.173)	**<0.001**	(-4.406, -2.200)
	healthy	adenoma	0.349	(-3.210, 1.149)	0.341	(-3.255, 1.141)
	normal	tumour	**<0.001**	(-8.857, -6.238)	**<0.001**	(-8.869, -6.251)
	normal	adenoma	**<0.001**	(-7.589, -3.014)	**<0.001**	(-7.618, -3.008)
	tumour	adenoma	0.065	(-0.138, 4.630)	0.066	(-0.155, 4.648)
Zhang EN AA	normal	healthy	**<0.001**	(-2.057, -0.759)	**<0.001**	(-2.043, -0.746)
	healthy	tumour	**0.038**	(0.046, 1.618)	**0.044**	(0.021, 1.595)
	healthy	adenoma	0.597	(-1.876, 1.086)	0.575	(-1.916, 1.070)
	normal	tumour	0.214	(-1.484, 0.332)	0.206	(-1.494, 0.322)
	normal	adenoma	**0.023**	(-3.350, -0.256)	**0.023**	(-3.375, -0.258)
	tumour	adenoma	0.133	(-2.835, 0.380)	0.135	(-2.850, 0.389)
EpiTOC AA	normal	healthy	**<0.001**	(-0.051, -0.041)	**<0.001**	(-0.051, -0.041)
	healthy	tumour	**<0.001**	(-0.069, -0.053)	**<0.001**	(-0.069, -0.053)
	healthy	adenoma	0.254	(-0.024, 0.006)	0.256	(-0.024, 0.006)
	normal	tumour	**<0.001**	(-0.116, -0.098)	**<0.001**	(-0.116, -0.098)
	normal	adenoma	**<0.001**	(-0.071, -0.039)	**<0.001**	(-0.071, -0.039)
	tumour	adenoma	**<0.001**	(0.036, 0.069)	**<0.001**	(0.036, 0.069)
HypoClock AA	normal	healthy	**<0.001**	(0.039, 0.047)	**<0.001**	(0.039, 0.047)
	healthy	tumour	**<0.001**	(0.052, 0.068)	**<0.001**	(0.052, 0.068)
	healthy	adenoma	0.064	(-0.001, 0.030)	0.063	(-0.001, 0.030)
	normal	tumour	**<0.001**	(0.094, 0.112)	**<0.001**	(0.094, 0.112)
	normal	adenoma	**<0.001**	(0.042, 0.073)	**<0.001**	(0.042, 0.073)
	tumour	adenoma	**<0.001**	(-0.063, -0.028)	**<0.001**	(-0.063, -0.028)
MiAge AA	normal	healthy	**<0.001**	(-839.781, -669.545)	**<0.001**	(-840.712, -670.554)
	healthy	tumour	**<0.001**	(-1,103.116, -823.647)	**<0.001**	(-1,101.641, -821.904)
	healthy	adenoma	0.342	(-501.540, 176.483)	0.346	(-499.797, 177.613)
	normal	tumour	**<0.001**	(-1,876.217, -1559.872)	**<0.001**	(-1,875.719, -1559.093)
	normal	adenoma	**<0.001**	(-1,263.955, -570.429)	**<0.001**	(-1,263.199, -570.251)
	tumour	adenoma	**<0.001**	(437.366, 1,164.340)	**<0.001**	(437.410, 1163.952)

Table S8. EAA differences between healthy ($n = 715$) and normal ($n = 505$) tissue from Dataset 2. p-values and 95% confidence interval were obtained from two-sample t-test. Significant differences ($p < 0.05$) are highlighted with bold text.

**EAA**	**Sex-adjusted**		**Unadjusted**	
	**p**	**95% CI**	**p**	**95% CI**
Horvath AA	0.566	(-0.434, 0.237)	0.441	(-0.467, 0.204)
Hannum AA	**<0.001**	(0.645, 1.750)	**<0.001**	(0.615, 1.727)
Pheno AA	**<0.001**	(0.587, 1.999)	**0.001**	(0.551, 1.983)
SkinBlood AA	0.056	(-0.014, 1.180)	0.050	(-0.001, 1.195)
PedBE AA	**0.001**	(0.066, 0.268)	**0.001**	(0.066, 0.267)
WuAA	**<0.001**	(0.062, 0.161)	**<0.001**	(0.061, 0.160)
Zhang BLUP AA	**0.002**	(0.281, 1.279)	**0.003**	(0.267, 1.267)
Zhang EN AA	**<0.001**	(0.603, 1.853)	**<0.001**	(0.577, 1.826)
EpiTOC AA	**0.022**	(0.000, 0.004)	**0.020**	(0.000, 0.004)
HypoClock AA	**<0.001**	(-0.004, -0.001)	**<0.001**	(-0.004, -0.001)
MiAge AA	**0.004**	(8.877, 45.999)	**0.003**	(9.434, 46.576)

Table S9. Coefficients of the fitted linear model (EA C̃A + sex), used in calculating EAA for the first part (for all healthy, normal and tumour samples). Fitted on n=716 healthy samples. For each model term (intercept, age and sex), the table contains coefficient β, standard error (SE), t-statistic and corresponding p-value. SexM - Sex (Male)

Clock	Intercept				Age				SexM			
	β	SE	t	p	β	SE	t	p	β	SE	t	p
Horvath	23.925	1.105	21.651	< 0.001	0.635	0.018	34.748	< 0.001	1.140	0.365	3.127	0.002
Hannum	39.919	1.908	20.918	< 0.001	0.439	0.032	13.907	< 0.001	1.648	0.630	2.616	0.009
Pheno	24.011	1.952	12.300	< 0.001	0.639	0.032	19.777	< 0.001	2.311	0.644	3.586	0.000
SkinBlood	31.502	1.369	23.014	< 0.001	0.663	0.023	29.252	< 0.001	-0.189	0.452	-0.418	0.676
PedBE	6.805	0.306	22.243	< 0.001	0.116	0.005	22.930	< 0.001	0.032	0.101	0.319	0.750
Wu	8.937	0.213	41.979	< 0.001	0.043	0.004	12.131	< 0.001	0.153	0.070	2.177	0.030
Zhang BLUP	39.675	1.261	31.471	< 0.001	0.571	0.021	27.360	< 0.001	0.560	0.416	1.345	0.179
Zhang EN	41.846	1.188	35.220	< 0.001	0.568	0.020	28.872	< 0.001	0.617	0.392	1.573	0.116
EpiTOC	0.108	0.009	11.483	< 0.001	0.001	0.000	6.971	< 0.001	-0.001	0.003	-0.289	0.772
HypoScore	0.872	0.004	200.53	< 0.001	0.000	0.000	-3.108	0.002	-0.003	0.001	-2.007	0.045
MiAge	1,144.41	116.12	9.855	< 0.001	14.43	1.922	7.510	< 0.001	-33.26	38.32	-0.868	0.386

Table S10. Coefficients of fitted linear model (EA C̃A + sex), used in calculating EAA for the second part (for all healthy and normal samples). Fitted on n=715 healthy samples. For each model term (intercept, age and sex), the table contains coefficient β, standard error (SE), t-statistic and corresponding p-value. SexM - Sex (Male)

Clock	Intercept				Age				SexM			
	β	SE	t	p	β	SE	t	p	β	SE	t	p
Horvath	23.669	1.102	21.482	< 0.001	0.640	0.018	35.083	< 0.001	1.083	0.363	2.983	0.003
Hannum	39.841	1.915	20.806	< 0.001	0.441	0.032	13.900	< 0.001	1.630	0.631	2.583	0.010
Pheno	23.443	1.939	12.090	< 0.001	0.649	0.032	20.209	< 0.001	2.183	0.639	3.416	0.001
SkinBlood	31.127	1.361	22.868	< 0.001	0.669	0.023	29.692	< 0.001	-0.273	0.449	-0.608	0.543
PedBE	6.735	0.305	22.077	< 0.001	0.117	0.005	23.230	< 0.001	0.016	0.101	0.163	0.871
Wu	8.929	0.214	41.797	< 0.001	0.043	0.004	12.128	< 0.001	0.151	0.070	2.147	0.032
Zhang BLUP	39.392	1.257	31.326	< 0.001	0.576	0.021	27.656	< 0.001	0.496	0.414	1.197	0.232
Zhang EN	41.642	1.188	35.048	< 0.001	0.571	0.020	29.044	< 0.001	0.571	0.392	1.459	0.145
EpiTOC	0.104	0.009	11.262	< 0.001	0.001	0.000	7.450	< 0.001	-0.002	0.003	-0.551	0.582
HypoScore	0.872	0.004	200.40	< 0.001	0.000	0.000	-3.243	0.001	-0.003	0.001	-1.914	0.056
MiAge	1,107.11	115.07	9.621	< 0.001	15.06	1.905	7.908	< 0.001	-41.65	37.92	-1.098	0.272

Table S11. Coefficients of fitted linear model (EA C̃A) and scaling parameters, used in calculating EAAs for the classifier. Linear model was fitted on $n = 341$ healthy samples. Standard Normal distribution scaling parameters were calculated on $n = 556$ samples.

Clock	Intercept				Age				Scaling parameters	
	β	SE	t	p	β	SE	t	p	mean	SD
Horvath	23.247	1.571	14.797	< 0.001	0.674	0.025	26.705	< 0.001	-0.067	4.651
Hannum	42.030	2.586	16.255	< 0.001	0.391	0.042	9.421	< 0.001	1.550	9.462
Pheno	23.452	2.845	8.243	< 0.001	0.653	0.046	14.293	< 0.001	3.199	10.792
SkinBlood	26.171	1.712	15.285	< 0.001	0.733	0.028	26.638	< 0.001	-0.523	5.423
PedBE	5.661	0.380	14.890	< 0.001	0.137	0.006	22.432	< 0.001	-0.340	1.298
Wu	9.140	0.312	29.268	< 0.001	0.043	0.005	8.500	< 0.001	-0.852	1.966
Zhang BLUP	36.325	1.524	23.834	< 0.001	0.617	0.024	25.191	< 0.001	-0.827	5.692
Zhang EN	42.729	1.620	26.374	< 0.001	0.569	0.026	21.846	< 0.001	-0.765	5.383
EpiTOC	0.049	0.010	4.817	< 0.001	0.002	0.000	10.871	< 0.001	-0.007	0.032
HypoScore	0.873	0.005	180.335	< 0.001	0.000	0.000	-2.343	0.020	0.003	0.020
MiAge	477.101	122.258	3.902	< 0.001	21.746	1.964	11.070	< 0.001	-120.004	440.154

Table S12. Folds for cross-validation. Folds in bold were also used for platform-dependent classifier

Fold	Train	Test
1	GSE101764, GSE149282, GSE142257, GSE166212	GSE132804_450k, GSE132804_epic
2	GSE101764, GSE132804_epic, GSE149282, GSE166212	GSE132804_450k, GSE142257
3	GSE101764, GSE132804_epic, GSE142257, GSE166212	GSE132804_450k, GSE149282
4	GSE101764, GSE132804_epic, GSE149282, GSE142257	GSE132804_450k, GSE166212
5	GSE132804_epic, GSE149282, GSE142257, GSE166212	GSE132804_450k, GSE101764
6	GSE101764, GSE149282, GSE132804_450k, GSE166212	GSE132804_epic, GSE142257
**7**	**GSE101764, GSE132804_450k, GSE142257, GSE166212**	**GSE132804_epic, GSE149282**
**8**	**GSE101764, GSE149282, GSE132804_450k, GSE142257**	**GSE132804_epic, GSE166212**
**9**	**GSE149282, GSE132804_450k, GSE142257, GSE166212**	**GSE132804_epic, GSE101764**
**10**	**GSE132804_epic, GSE149282, GSE132804_450k, GSE166212**	**GSE101764, GSE142257**
**11**	**GSE101764, GSE132804_epic GSE132804_450k, GSE166212**	**GSE142257, GSE149282**
**12**	**GSE101764, GSE132804_epic, GSE149282, GSE132804_450k**	**GSE142257, GSE166212**

Table S13. Models Coefficients and Performance

**Model**	Main classifier: $\alpha = 0.05, \lambda = 0.16$	Parameters: $\alpha = 0.25, \lambda = 0.25$	Parameters: $\alpha = 0.1, \lambda = 0.35$	Platform ID included: $\alpha = 0.05, \lambda = 0.68$
**Coefficients**				
(Intercept)	-0.7387	-0.5943	-0.7094	-0.5677
Horvath	-	-	-	-
Hannum	0.1601	0.0017	-	0.0641
Pheno	0.5132	0.3044	0.5983	0.1729
SkinBlood	-0.0408	-	-	-
PedBE	-0.1533	-0.0888	-0.1191	-0.0881
Wu	-0.5147	-0.4272	-0.7161	-0.2179
Zhang BLUP	-0.0416	-	-	-0.0190
Zhang EN	-0.1423	-	-0.0814	-0.0322
EpiTOC	-0.0351	-	-	-0.0426
HypoScore	0.1679	0.0212	0.1256	0.0438
MiAge	-0.1342	-0.0756	-0.0602	-0.0865
sex	0.4649	0.2288	0.4171	0.1944
**Performance**				
ROC-AUC	0.8858	0.8817	0.8351	0.9207
ROC-AUC 95%CI	[0.8497, 0.9218]	[0.8449, 0.9185]	[0.7910, 0.8793]	[0.8922, 0.9492]

1.2 Supplementary Figures

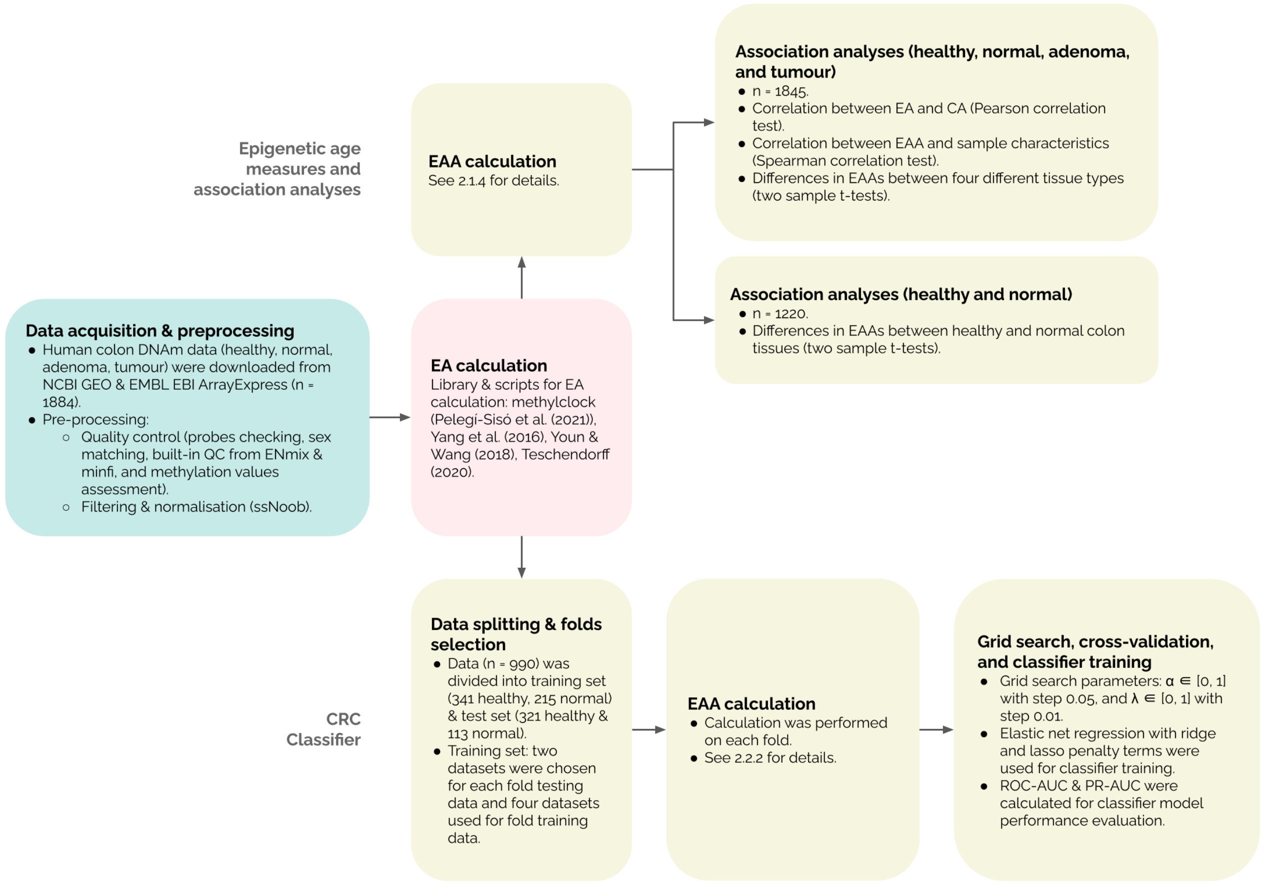

Figure S1. Graphical summary of methodology. Abbreviations: CA - Chronological age, DNAm - DNA methylation, EA - Epigenetic age, EAA - Epigenetic age acceleration, F - Female, M - Male, QC - Quality control.

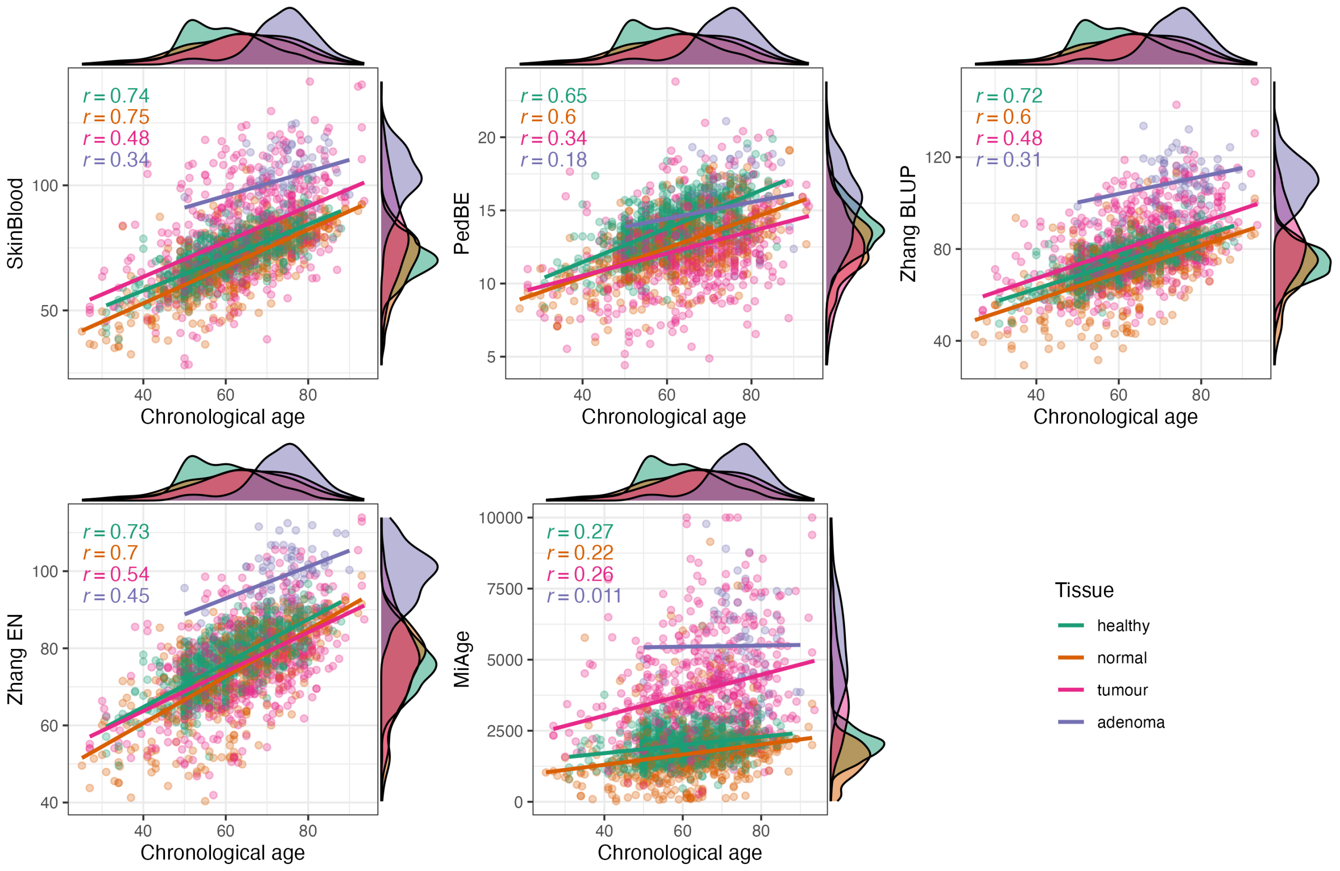

Figure S2. Correlation between chronological age and epigenetic age estimates in four different tissues (healthy (n=716), normal (n=522), tumour (n=535), and adenoma (n=72)) based on Pearson correlation test. Different colours represent different tissues.

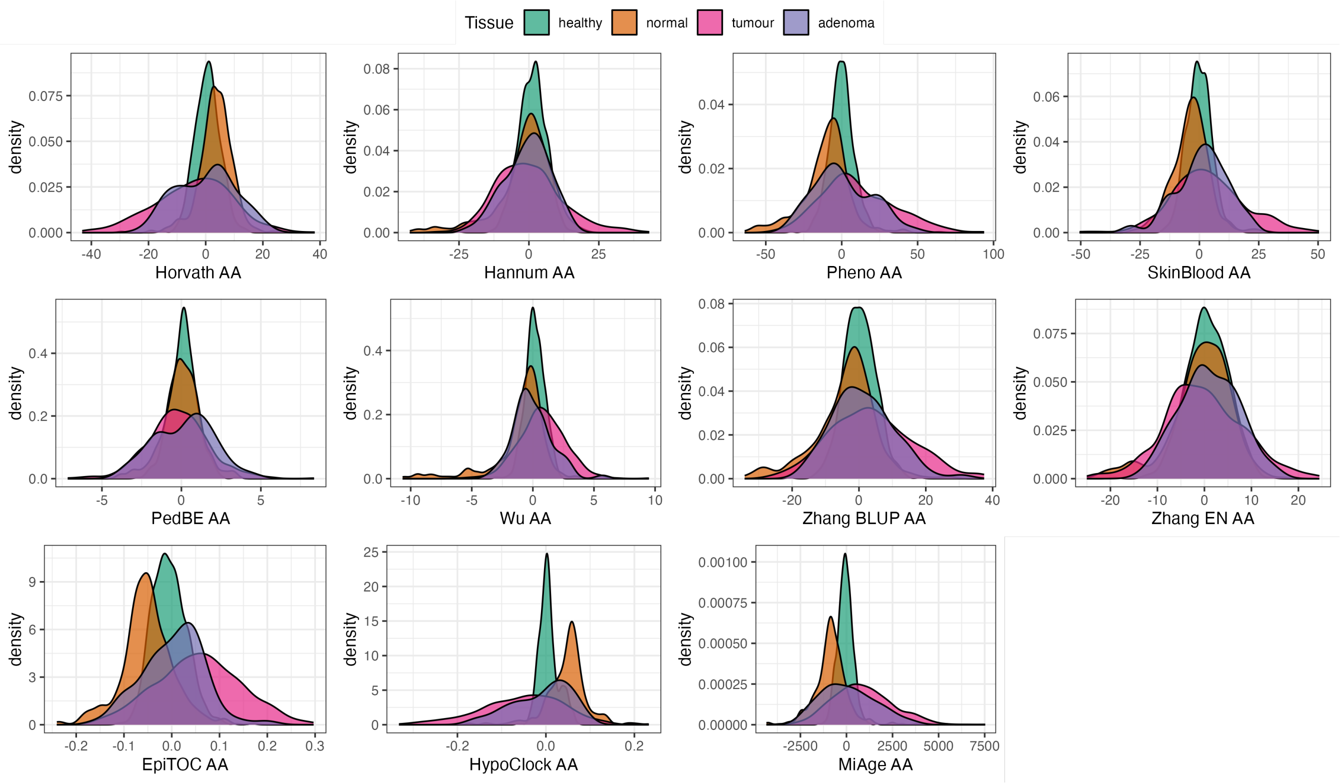

Figure S3. Density plots of EAAs distribution in four different tissues (healthy, normal, tumour, adenoma) from Dataset 1.

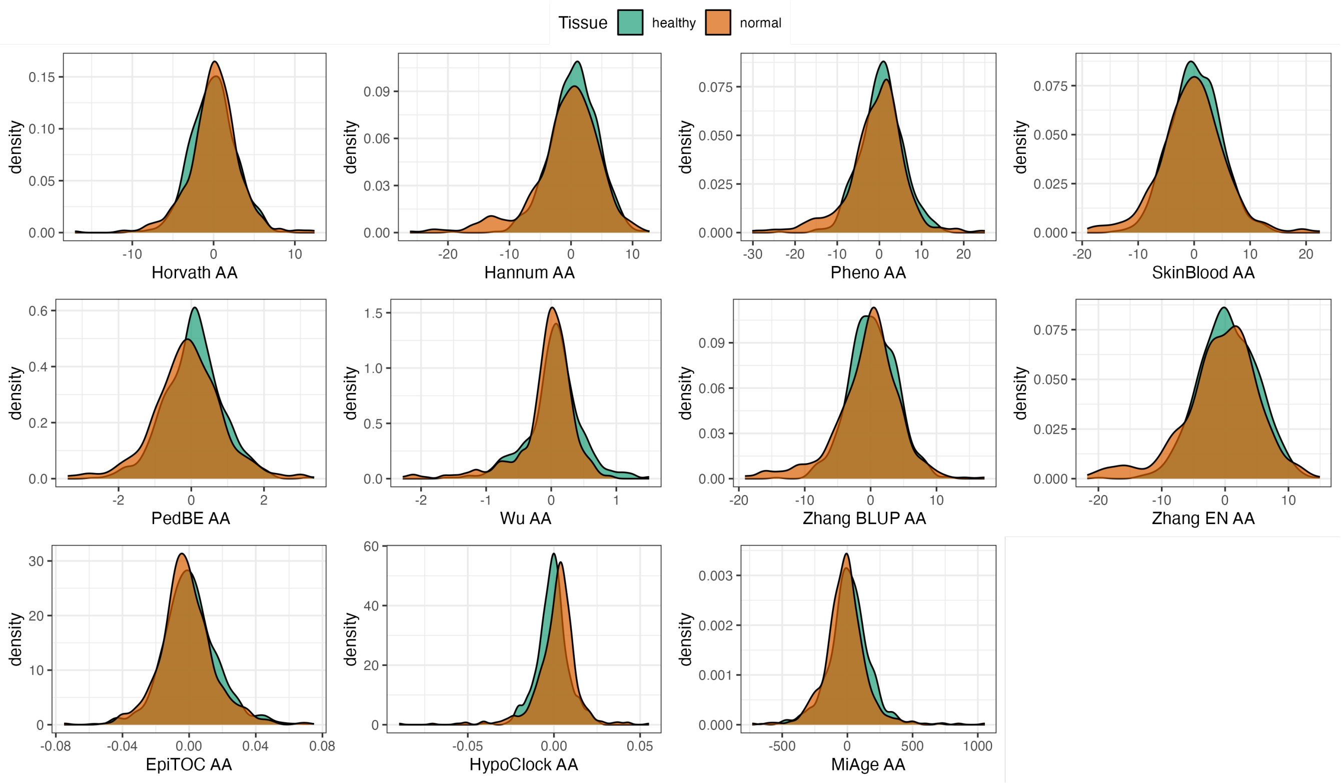

Figure S4. Density plots of EAAs distribution in healthy and normal tissues from Dataset 2.

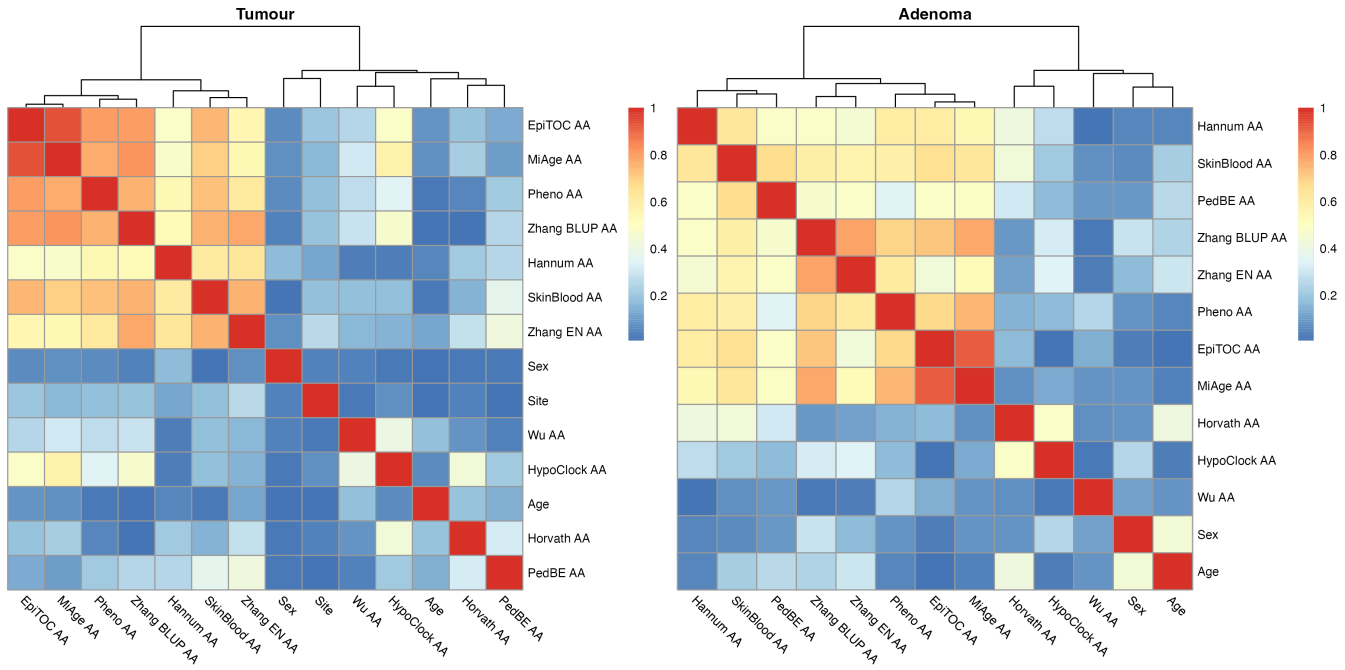

Figure S5. Heatmap of Spearman correlation between sample characteristics and sex-adjusted epigenetic age accelerations (EAAs) in tumour and adenoma tissues from Dataset 1. Correlation coefficients are in absolute values.

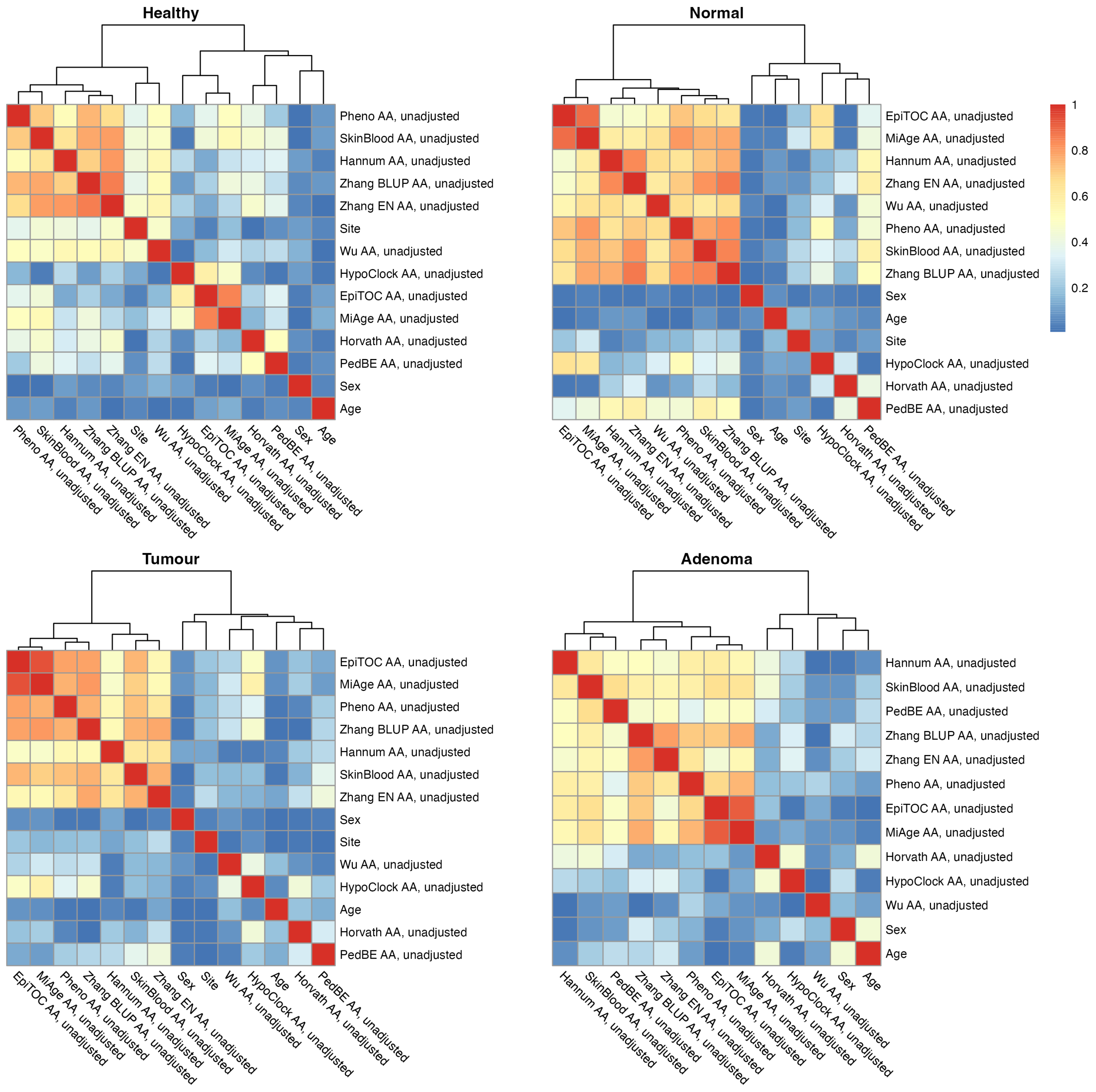

Figure S6. Heatmap of Spearman correlation between sample characteristics and unadjusted epigenetic age accelerations (EAAs) in four different tissues (healthy, normal, tumour, and adenoma) from Dataset 1. Correlation coefficients are in absolute values.

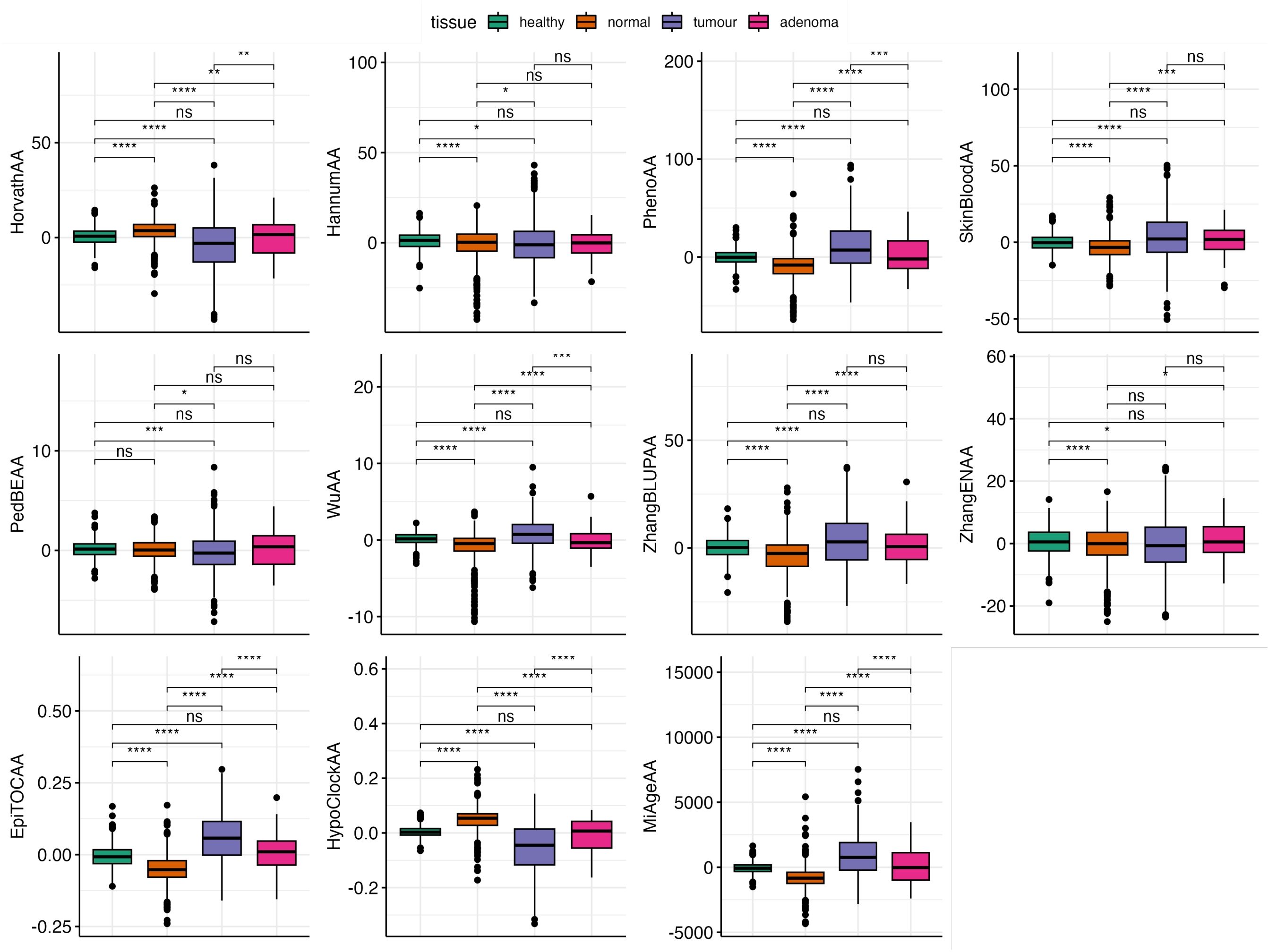

Figure S7. Boxplot of sex-adjusted EAAs in four different tissues from Dataset 1. The p-values were obtained from Welch's two-sample t-test. *p<0.05, ** p<0.001, ***p<0.001, ****p<0.0001.

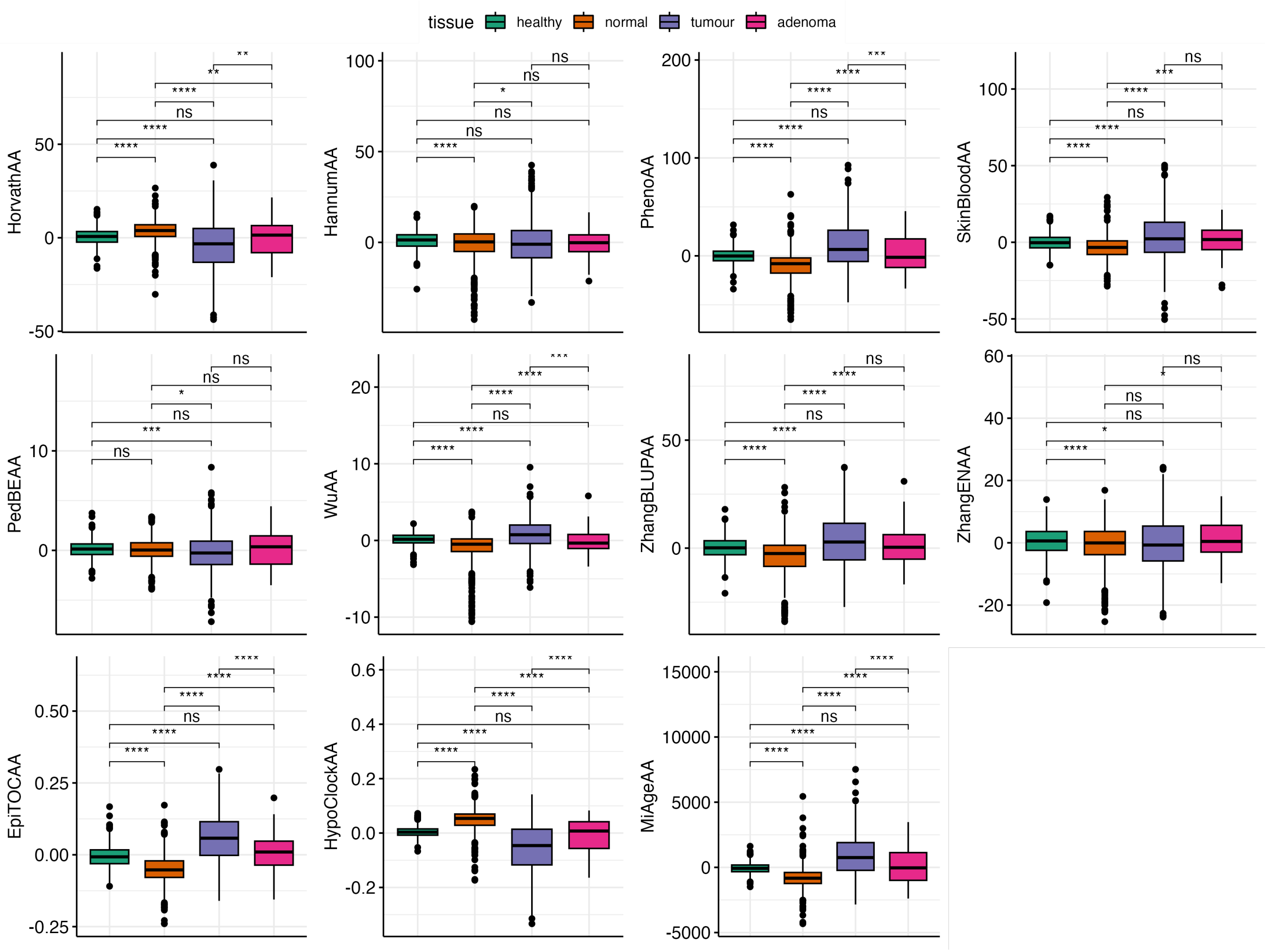

Figure S8. Boxplot of unadjusted EAAs in four different tissues from Dataset 1. The p-values were obtained from Welch's two-sample t-test. *p<0.05, ** p<0.001, ***p<0.001, ****p<0.0001.

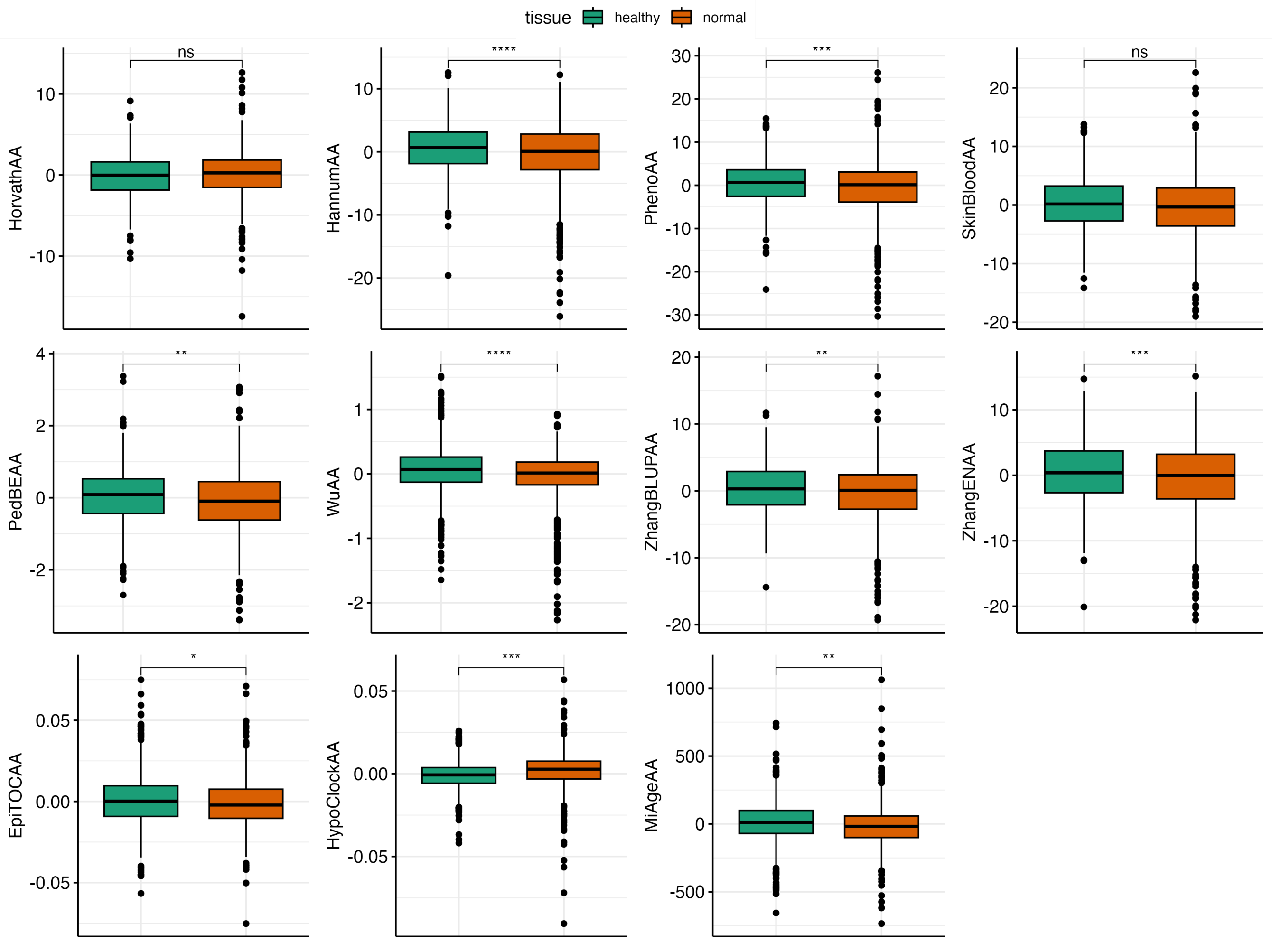

Figure S9. Boxplot of unadjusted EAAs in healthy and normal tissues from Dataset 2. The p-values were obtained from Welch's two-sample t-test. *p<0.05, ** p<0.001, ***p<0.001, ****p<0.0001.

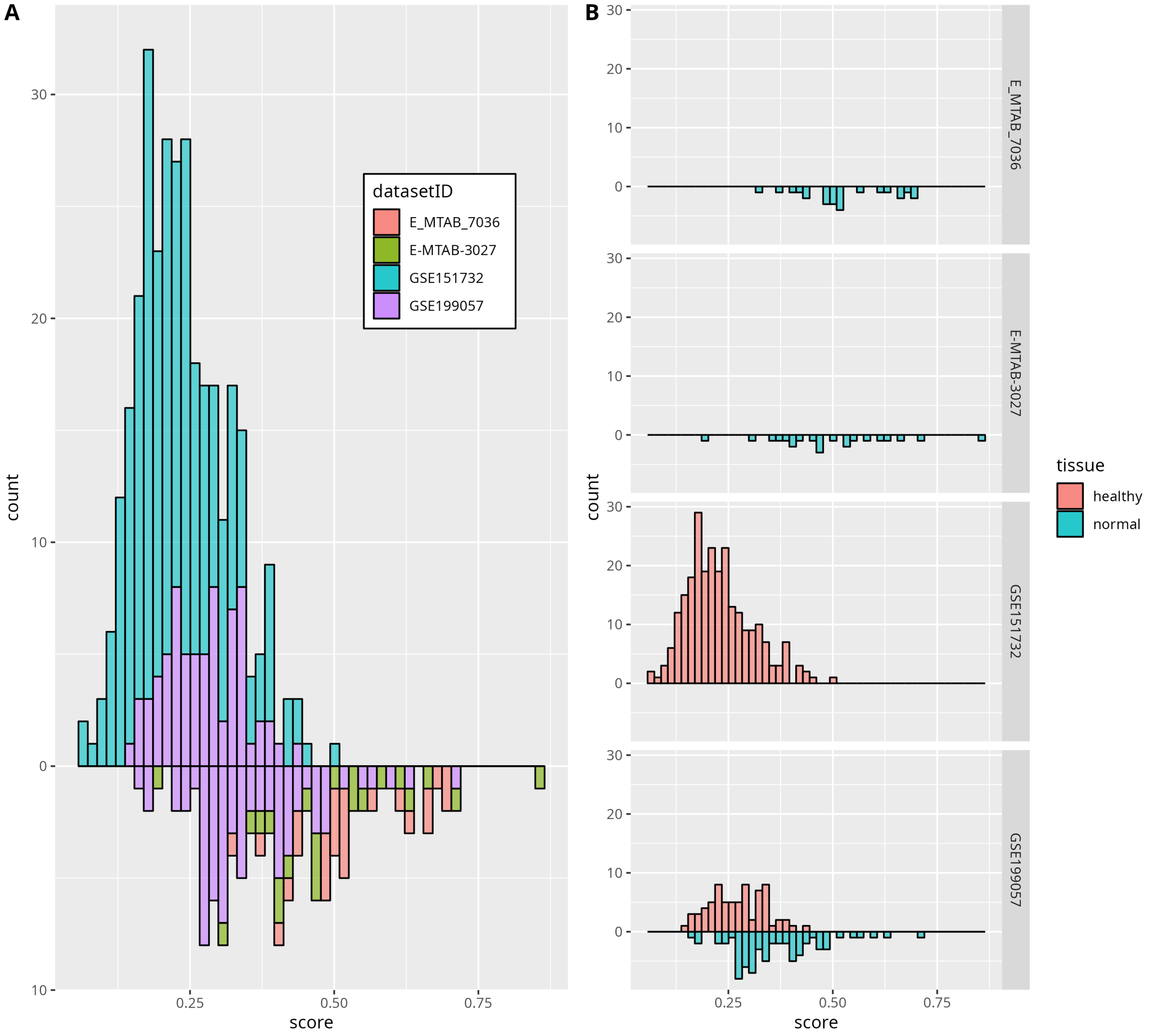

Figure S10. Classifier scores histograms for all testing data coloured by dataset (A), and for each dataset separately (B)

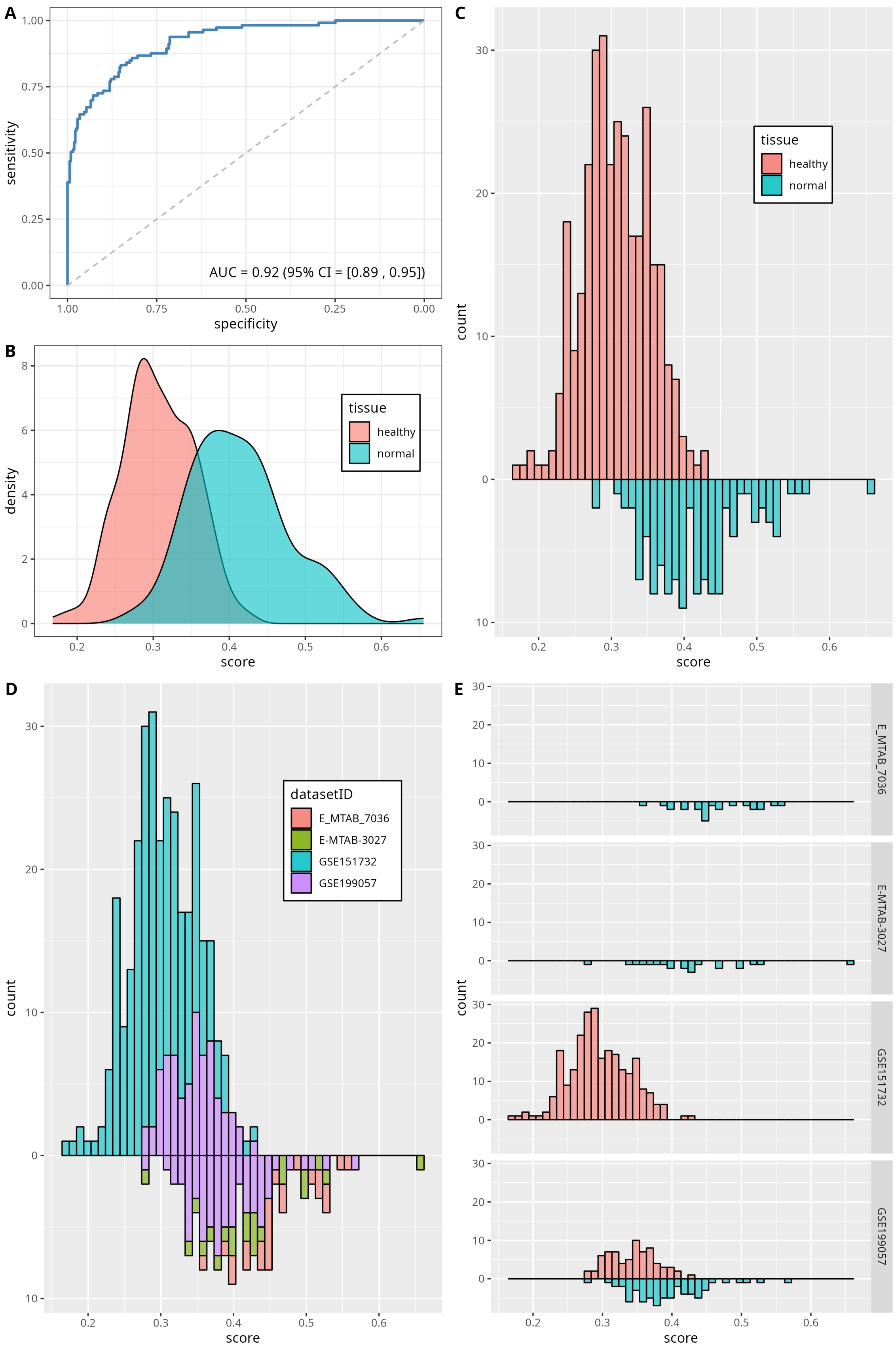

Figure S11. Platform-dependent classifier performance. ROC curve (A), density plot (B), scores histograms for all testing data coloured by tissue (C) and dataset ID (D), and for each dataset separately (E)

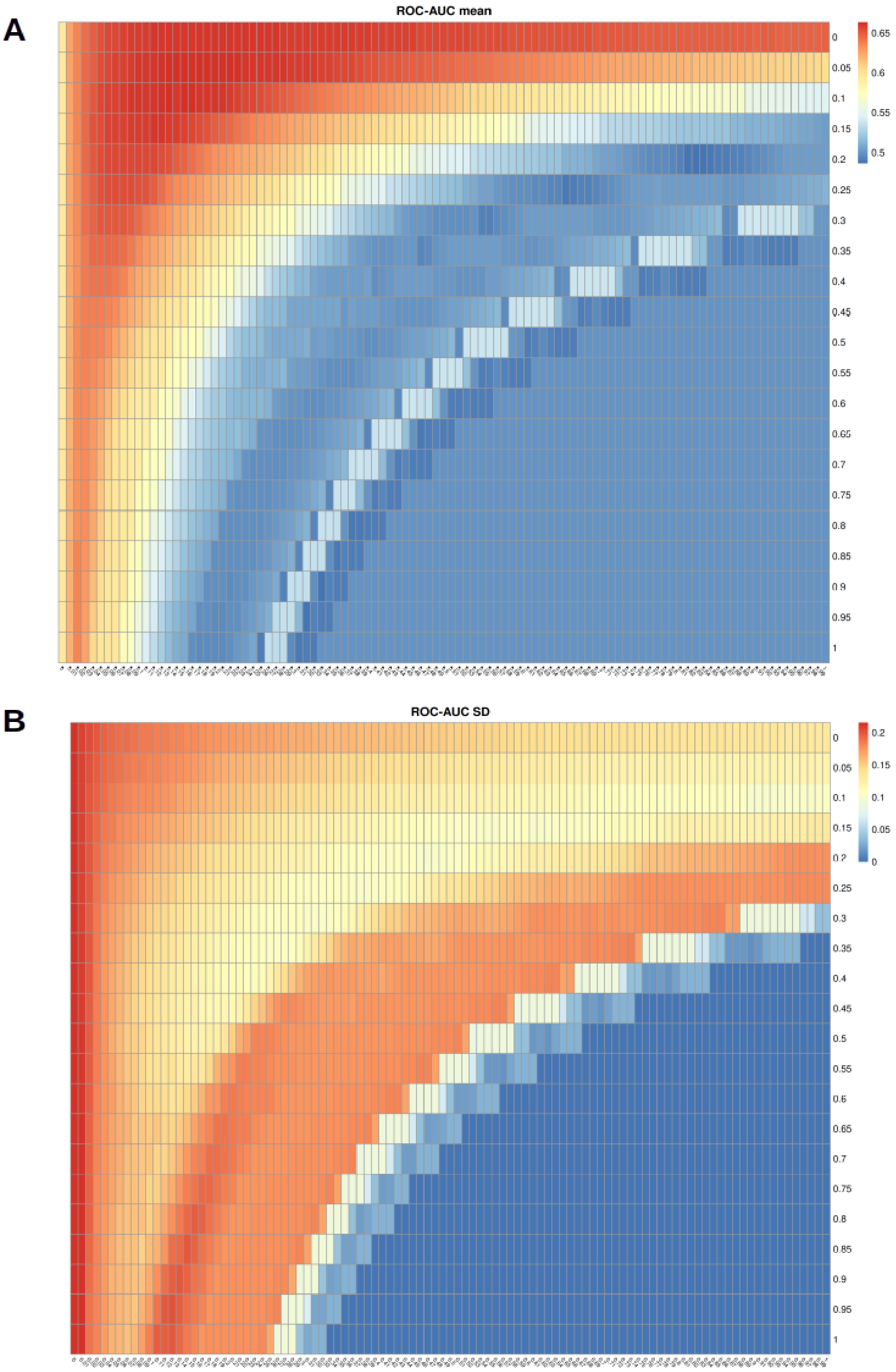

Figure S12. Heatmaps for the average ROC-AUC means (A) and standard deviations (B) measures for each pair of parameters α (y-axis) and λ (x-axis) across twelve cross-validation folds.
